# Etch–Bleach–Seal Technique in Managing Enamel Hypomineralization: A Case Report With Narrative Literature Review

**DOI:** 10.1155/crid/6693068

**Published:** 2026-05-11

**Authors:** Matine Gharavi, Sara Tavassoli-Hojjati, Zahra Abdolahpour

**Affiliations:** ^1^ Department of Pediatric, Faculty of Dentistry, Tehran Medical Sciences, Islamic Azad University, Tehran, Iran, azad.ac.ir

**Keywords:** case report, dental enamel hypomineralization, etch–bleach–seal, ICON infiltration, sodium hypochlorite

## Abstract

**Background:**

Molar incisor hypomineralization (MIH) opacities significantly affect pediatric patients′ esthetics and psychosocial well‐being. This case is unique as it demonstrates the clinical efficacy of a modified etch–bleach–seal protocol, utilizing a double application of sodium hypochlorite to address deep pigmentations where standard conservative approaches may be limited.

**Objective:**

This study is aimed at evaluating the clinical efficacy and existing literature supporting the etchâ€“bleachâ€“seal technique, a minimally invasive approach developed by Wright for managing enamel hypomineralizationâ€“related opacities.

**Case Description:**

A 10‐year‐old female presented with esthetic concerns regarding yellow‐brown discoloration on her upper central incisors. After clinical and radiographic evaluations, a diagnosis of MIH was established. The patient was treated using the etch–bleach–seal technique, including phosphoric acid etching, sodium hypochlorite bleaching, and ICON resin sealing.

**Outcomes:**

Posttreatment, the patient showed significant improvement in enamel appearance. A follow‐up session at 3 months led to further enhancement of esthetic results.

**Conclusion:**

The modified etch–bleach–seal protocol appears to be a promising conservative option for deep MIH opacities. However, as this represents a single clinical case, longitudinal studies are warranted to confirm its long‐term durability and impact on enamel integrity.

## 1. Introduction

The biological synthesis of dental enamel is a complex process orchestrated by highly specialized cells known as ameloblasts. This intricate formation involves an initial secretory stage, during which key matrix proteins such as ameloblastin, amelogenin, and enamelin are deposited, followed by critical phases of mineralization and maturation [1]. Structurally, mature enamel is distinguished by its exceptional mineral density, primarily composed of densely organized hydroxyapatite crystallites that account for 95% of its total weight and 87% of its volume. The remaining composition consists of water and organic matter, a unique organization that grants enamel its remarkable hardness, establishing it as the most resilient tissue in the human body [2].

Despite its durability, developmental enamel defects are frequently encountered in both primary and permanent dentitions. Genetic predispositions or environmental stressors can disrupt odontogenesis, leading to clinical conditions such as hypoplasia or hypomineralization [3]. While hypoplasia stems from disturbances during the secretory phase, hypomineralization typically occurs due to interruptions during the subsequent mineralization or maturation stages. Identifying the precise etiology of these anomalies often presents a clinical challenge [4]. Beyond the physical presentation, the psychological consequences of enamel defects in pediatric patients are profound. Dental esthetics play a pivotal role in social perception, and affected children may face teasing or negative judgments based on their dental appearance, which can significantly impact their social well‐being [5].

In response to these esthetic and psychological challenges, minimally invasive interventions are increasingly sought. This paper is aimed at introducing the etch–bleach–seal technique as a conservative management strategy for enamel opacities associated with hypomineralization. Furthermore, this report evaluates the efficacy of a specific modification of this protocol. The following case describes the application of this modified etch–bleach–seal method in a 10‐year‐old patient exhibiting prominent anterior discoloration. By presenting this real‐world application alongside a targeted literature review, this manuscript seeks to illustrate a conservative pathway toward achieving esthetically satisfactory outcomes while preserving natural tooth structure in young individuals.

## 2. Materials and Methods

This manuscript was designed as a narrative review rather than a formal systematic review. To provide a transparent overview of the existing literature on the etch–bleach–seal technique for enamel hypomineralization, a structured search was conducted across three major electronic databases: PubMed, Google Scholar, and Scopus. The search covered articles published between 2000 and 2023, and only articles in English were considered. The following keywords were used in various combinations: “dental enamel hypomineralization,” “enamel discoloration,” “etch–bleach–seal,” “tooth bleaching agents,” “sodium hypochlorite,” and “molar incisor hypomineralization (MIH).” Initially, 729 articles were identified. Manual reference checks of selected articles were also performed to identify additional relevant studies.

**Figure 1:**
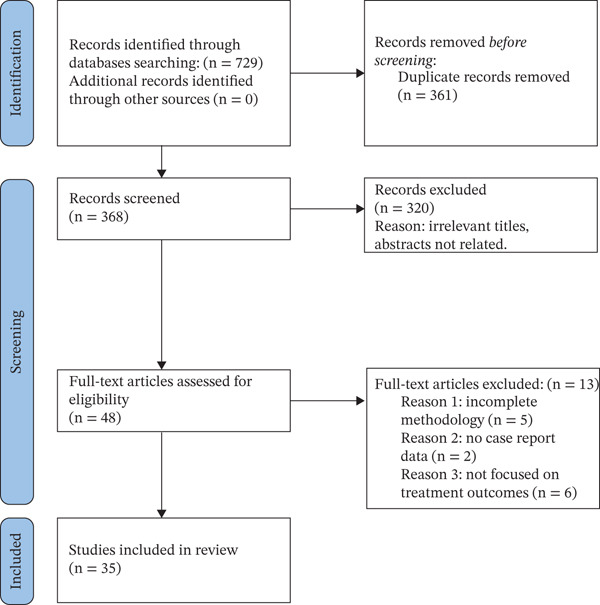
PRISMA‐style flow diagram illustrating the literature search and selection process. The diagram is provided to improve transparency in documenting the identification and screening of relevant publications. However, this study was conducted as a narrative review rather than a formal systematic review, and no formal risk‐of‐bias assessment or systematic, quantitative synthesis of the included studies was undertaken.

Inclusion criteria encompassed case reports involving enamel discoloration treated using the etch–bleach–seal method or closely related minimally invasive protocols; narrative reviews focused on enamel hypomineralization, developmental enamel defects, or conservative treatment approaches, including etch–bleach–seal or similar techniques; and clinical or experimental studies evaluating bleaching or microabrasion strategies that included sodium hypochlorite or the etch–bleach–seal protocol, particularly in the context of molar incisor hypomineralization (MIH) or fluorosis. Articles that were not in English, not peer‐reviewed, duplicates, or unrelated to the etch–bleach–seal technique were excluded. After applying selection criteria and removing duplicates, 35 articles met the final inclusion criteria, as illustrated in Figure 1. This flow diagram is presented solely to enhance transparency in reporting the literature selection process, in line with common reporting standards; no formal risk‐of‐bias assessment or systematic, quantitative synthesis of the included studies was performed, and the present work should be interpreted as a narrative review.

In the present narrative review, the available literature on managing enamel hypomineralization with the etch–bleach–seal approach is predominantly based on low‐level evidence. Most of the identified publications consist of single case reports and a limited clinical case series, complemented by several narrative reviews describing conservative management strategies for hypomineralized enamel. No prospective controlled clinical trials or in vitro experimental studies specifically evaluating the etch–bleach–seal technique for MIH were identified in our search. This distribution of study designs was taken into account when interpreting the findings of the review.

Articles that were not in English, not peer‐reviewed, duplicates, or unrelated to the etch–bleach–seal technique were excluded. After applying selection criteria and removing duplicate articles, 35 articles met the final inclusion criteria (Figure 1).

### 2.1. Ethical Considerations

This case report adhered to the ethical principles of the Declaration of Helsinki. No institutional ethical approval was required, as the treatment provided fell within the scope of routine pediatric dental care.

### 2.2. Patient Consent Statement

Written informed consent was obtained from the patient′s legal guardians for both the clinical treatment and the publication of all case‐related information, including clinical photographs.

### 2.3. Patient Information

A 10‐year‐old female presented with concerns regarding the esthetic appearance of her anterior teeth. Her parents reported no familial history of dental anomalies, and her medical, prenatal, birth, and psychosocial histories were unremarkable. Primary dentition had developed normally, and no prior dental interventions were recorded.

### 2.4. Clinical Findings

On clinical examination, the patient exhibited demarcated opacities with a yellow‐brown appearance, particularly on the upper central incisors. There were no signs of cavitation or posteruptive enamel breakdown (Table 1).

**Table 1:** Timeline of the patient’s clinical history and treatment.

Time point	Event
Age 8	Parents first noticed discoloration
Age 10	Presented for consultation
Day 0	Diagnosis confirmed, and treatment plan discussed
Day 7	Etch–bleach–seal procedure performed
Month 3	Follow‐up and second application of treatment

### 2.5. Diagnostic Assessment

Differential diagnoses considered included dental fluorosis, amelogenesis imperfecta, and traumatic hypoplasia.

Fluorosis was ruled out due to the absence of a history of excessive fluoride exposure and the well‐demarcated nature of the opacities rather than the diffuse presentation seen in fluorosis.

Amelogenesis imperfecta was excluded given the absence of similar lesions in the primary dentition, lack of familial pattern, and no generalized enamel defects.

Traumatic hypoplasia was also unlikely as there was no history of trauma to the primary incisors.

Periapical radiographs were taken to rule out other enamel or dentin abnormalities. No radiolucencies or pulp involvement was observed.

### 2.6. Diagnosis

After a thorough case history, clinical assessment, evaluation of differential diagnoses, and radiographic confirmation of normal underlying structures, a final diagnosis of enamel hypomineralization affecting the permanent upper central incisors was made. The prognosis for esthetic improvement and maintenance of enamel integrity was considered favorable, given the intact surface of the enamel and the conservative nature of the proposed intervention.

### 2.7. Therapeutic Intervention: Treatment Plan

Considering the patient′s young age, esthetic concerns, and desire to avoid invasive procedures, several treatment options were discussed, including microabrasion, in‐office bleaching, composite veneers, and the etch–bleach–seal technique.

Microabrasion was deemed insufficient due to the depth of discoloration.

Composite veneers were considered too invasive for a 10‐year‐old with partially developed dentition.

In‐office bleaching posed risks of sensitivity and inconsistent outcomes.

Based on the literature, the patient′s cooperation, and long‐term esthetic potential, the etch–bleach–seal technique was selected as a conservative, safe, and effective option.

The treatment plan was thoroughly explained to the patient and her parents, and informed consent was obtained. The treatment followed a modified protocol as described by MacLean which was adapted to address the specific depth and color of the lesions in this patient.

#### 2.7.1. First Step: Tooth Preparation

**Figure 2:**
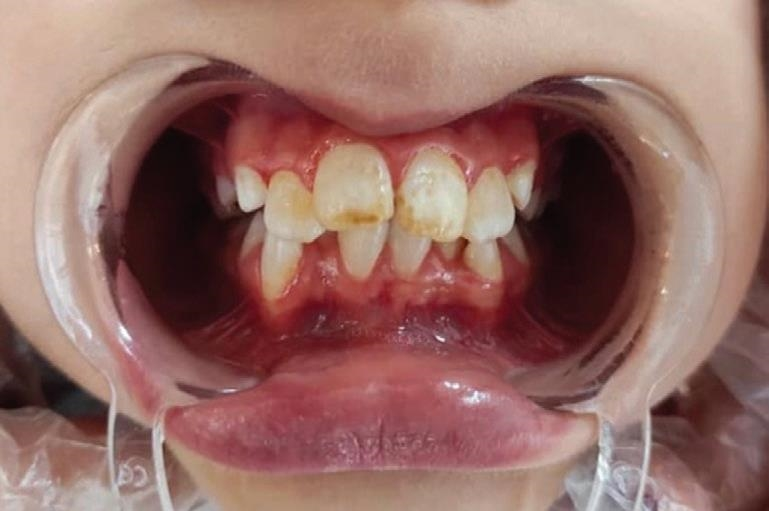
Before treatment.

The teeth were cleaned with pumice and water. Isolation was achieved using cotton rolls and high‐volume suction. Although rubber dam isolation is generally preferred for adhesive procedures, cotton roll isolation was selected in this case due to the limited cooperation of the pediatric patient and the relatively short duration of the procedure (Figure 2).

#### 2.7.2. Second Step: Etch and Bleach

**Figure 3:**
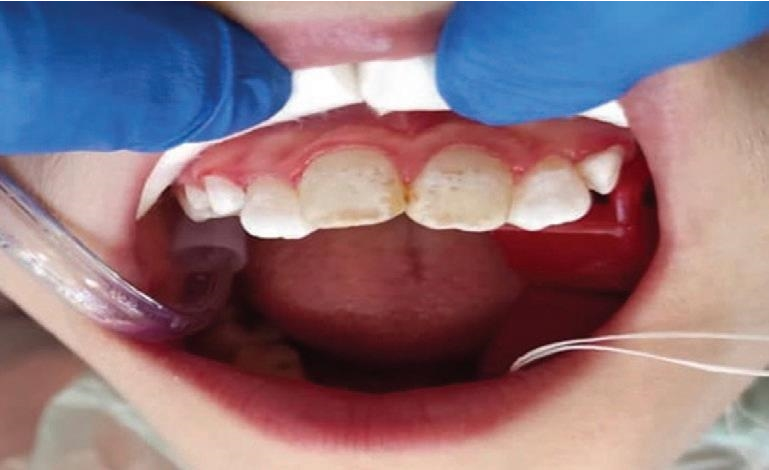
After the first time of etching.

The enamel surface was etched with 37% phosphoric acid (Meta Etchant by Meta Biomed, Korea) for 2 min to increase enamel porosity and enhance penetration of the bleaching agent (Figure 3).

**Figure 4:**
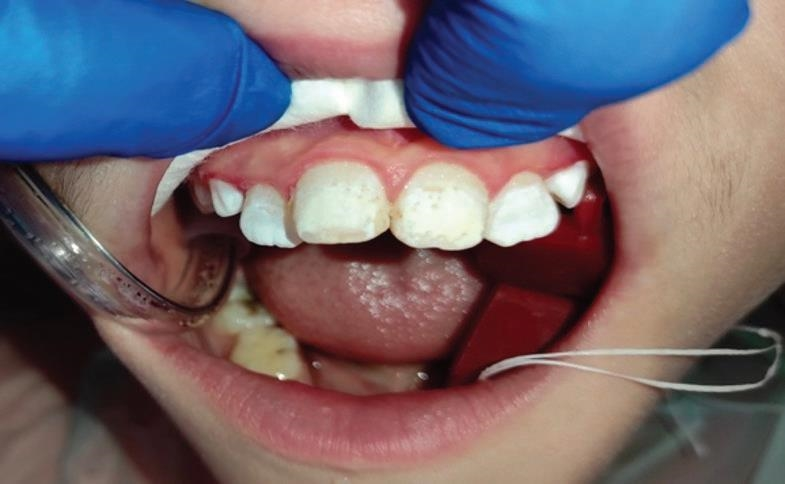
After the second time of etching.

Following the initial rinse and dry, the enamel was re‐etched for another 2 min, again rinsed and dried to ensure consistent surface conditioning (Figure 4).

**Figure 5:**
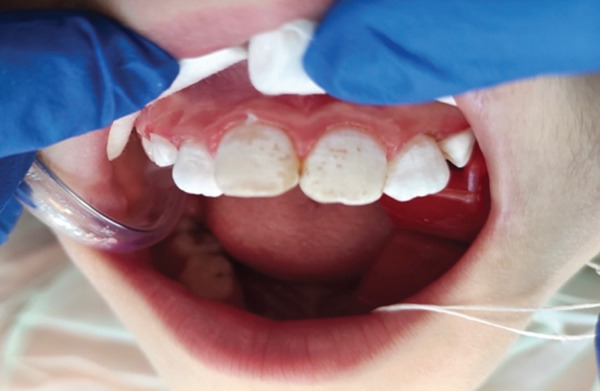
After the first time of applying sodium hypochlorite.

A 5.25% sodium hypochlorite solution (EX Hype by Parla, Iran) was applied using a microbrush directly to the surface of the defect (Figure 5).

**Figure 6:**
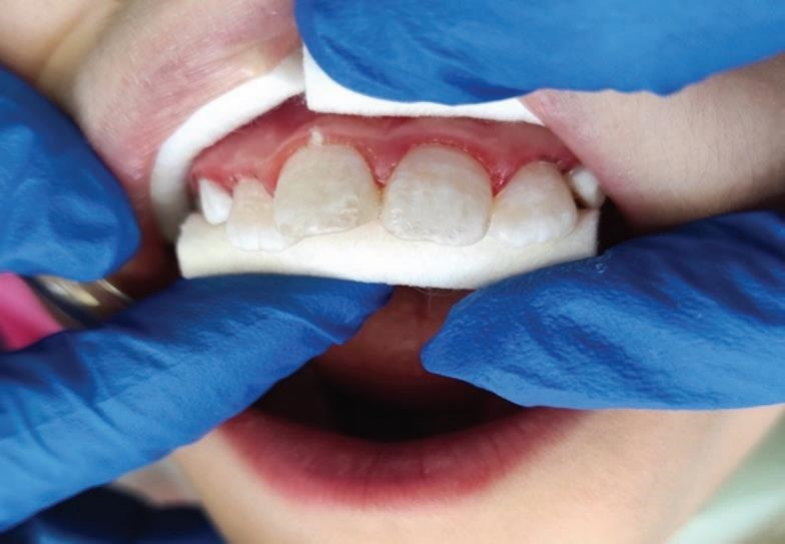
After the second time of applying sodium hypochlorite.

The bleaching process was maintained for 15 min. A second application of sodium hypochlorite was subsequently performed to enhance esthetic results. This step is aimed at locally bleaching a vital tooth by removing the colored proteins responsible for the yellow‐brown opacities in hypomineralized enamel (Figure 6).

#### 2.7.3. Third Step: Etch and Seal

**Figure 7:**
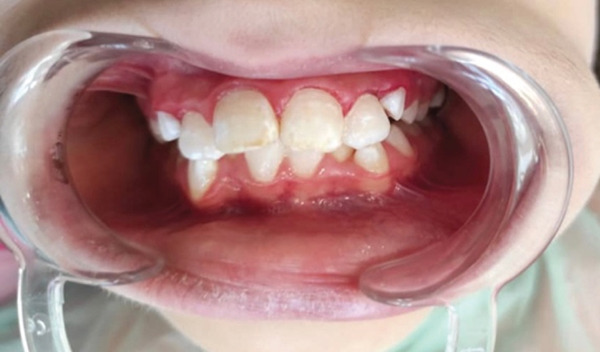
After the final step of ICON.

After achieving optimal bleaching, the enamel surface was rinsed and dried to remove all bleaching agents. A resin infiltrant (Icon, DMG, Hamburg, Germany) was used to seal the etched and bleached enamel. This was performed to prevent re‐entry of organic material into the porous, hypomineralized surface. The sealant was applied and light‐cured for 20 s, effectively stabilizing the treated enamel (Figure 7).

### 2.8. Follow‐Up and Outcomes

**Figure 8:**
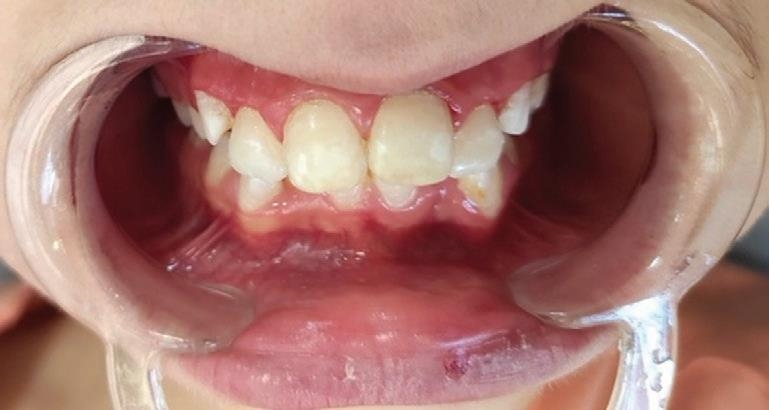
After repeating the procedure in the follow‐up session.

In pediatric dental care, age, enamel maturity, and patient cooperation play critical roles in the success of minimally invasive treatments. At the 3‐month follow‐up, clinical evaluation showed significant improvement in the color and translucency of the treated teeth. There were no signs of sensitivity, surface damage, or relapse. The patient and her parents expressed high satisfaction with the results. To enhance the esthetic outcome further, the same bleaching and sealing protocol was repeated during this visit. No adverse events or posttreatment complications were observed throughout the follow‐up period. In addition to the clinical success, the patient and her parents expressed high satisfaction with the esthetic results and the conservative, pain‐free nature of the procedure, which avoided the need for more invasive restorative treatments (Figure 8).

### 2.9. Patient Perspective

The patient′s parents expressed significant satisfaction with the esthetic outcome of the clinical procedure. They noted that the previously prominent yellow‐brown discolorations on their daughter′s upper central incisors had caused her to feel self‐conscious when smiling. According to the parents, the noninvasive nature of the etch–bleach–seal technique was highly appreciated, as it avoided the need for local anesthesia or aggressive tooth preparation. Following the successful completion of the treatment protocol and the 3‐month follow‐up, they observed a substantial improvement in the child′s self‐confidence, reporting that the final esthetic appearance of the teeth was much better than they had initially anticipated.

### 2.10. Literature Review

A total of 35 relevant articles were retained for descriptive synthesis in this narrative review, providing an overview of conservative treatment strategies for enamel hypomineralization. However, the overall evidence base is largely composed of low‐level and heterogeneous study designs. Most of the included publications are single case reports describing the use of the etch–bleach–seal protocol or similar minimally invasive approaches in individual patients, supplemented by one small, noncomparative clinical case series and several narrative reviews on developmental enamel defects and MIH. In addition, one in vitro randomized controlled trial on extracted MIH‐affected teeth and one randomized clinical trial conducted in children with nonpitted fluorotic stains rather than MIH provide only preliminary comparative data regarding bleaching and microabrasion protocols. No prospective randomized controlled clinical trials specifically assessing the etch–bleach–seal technique in MIH were identified. Given this distribution of study designs, the conclusions drawn from the available literature must be interpreted with caution, and the apparent clinical benefits should be viewed as preliminary rather than definitive.

Within these limitations, the available literature, primarily consisting of single case reports and one small, noncomparative clinical case series, suggests that the etch–bleach–seal approach can yield promising but preliminary esthetic improvements in selected cases of MIH‐related and fluorotic discolorations. These reports, while illustrating clinical feasibility, lack control groups and extended follow‐up, thus providing low‐level evidence. Several publications emphasized the importance of combining sodium hypochlorite bleaching with subsequent resin infiltration to stabilize esthetic results and minimize the risk of restaining; however, these observations are also derived from case‐based evidence.

Regarding comparative data, one in vitro randomized controlled trial on extracted MIH‐affected teeth (Gandhi et al.) investigated the effect of sodium hypochlorite deproteinization on resin penetration, yielding preliminary experimental insights rather than robust clinical outcome data. Furthermore, one randomized clinical trial in children with nonpitted fluorotic stains (Singhal et al.) compared different bleaching and microabrasion protocols, including the etch–bleach–seal method, but this study offers only preliminary comparative data and did not focus specifically on MIH. Consequently, the evidence comparing the etch–bleach–seal technique directly against other interventions in MIH patients is limited and preliminary.

Taken together, these findings indicate potential benefits but are still characterized by small sample sizes and designs that restrict their generalizability. The overall strength of the available evidence supporting the etch–bleach–seal technique in MIH remains low, necessitating cautious interpretation of reported outcomes.

In comparative studies, the etch–bleach–seal method has been described as a conservative option compared to microabrasion, peroxide‐based bleaching, and composite restorations, although direct comparative clinical evidence remains limited.

Moreover, the literature demonstrated a growing interest in combining minimally invasive techniques to manage developmental enamel defects, particularly in younger patients where both tissue conservation and esthetic outcome are critical.

## 3. Discussion

The primary strength of this case report lies in the application of a modified, minimally invasive protocol (the etch–bleach–seal technique) that successfully managed deep enamel opacities while preserving natural tooth structure. Furthermore, the 3‐month follow‐up demonstrates the immediate stability of the esthetic outcome. However, as this is a single case report, its findings cannot be broadly generalized to all MIH patients. Longitudinal clinical trials with larger sample sizes are necessary to establish long‐term success rates and refine the protocol for various lesion depths.

In addition, the supporting literature on the etch–bleach–seal technique is largely composed of single case reports, one small, noncomparative clinical case series in MIH patients, and a limited number of narrative reviews, together with one in vitro randomized controlled trial on extracted MIH‐affected teeth (Gandhi et al.) and one preliminary randomized clinical trial in children with nonpitted fluorotic stains (Singhal et al.). Although these studies provide preliminary insights, they do not amount to robust high‐level evidence, and none represents a prospective randomized clinical trial directly assessing the etch–bleach–seal protocol in MIH patients. Overall, this body of evidence still corresponds to a low level of certainty, restricts the generalizability of the findings, and necessitates cautious interpretation.

This case underscores the importance of conservative, minimally invasive treatment in a pediatric patient presenting with anterior enamel discoloration due to hypomineralization.

### 3.1. Hypomineralization

Enamel hypomineralization is characterized by a qualitative impairment in the mineral density of the enamel, even though the original tissue thickness remains unchanged. This condition results in altered translucency, which may cause the tooth surface to appear opaque or creamy‐white or exhibit distinct yellow‐to‐brown pigmentations. These opacities are typically categorized into either demarcated or diffuse patterns [6]. Furthermore, hypomineralized defects are classified as either localized or generalized; focal lesions may be triggered by factors such as trauma, radiation exposure, or infections involving the primary precursors. Conversely, widespread defects are frequently associated with broader environmental, genetic, or systemic influences. If these systemic stressors occur during specific windows of tooth development, they can lead to chronological hypomineralization, affecting only the specific teeth undergoing formation during that timeframe [7].

According to recent investigations, the global occurrence of MIH is estimated to be between 12.9% and 14.2%, though these figures fluctuate significantly across different geographical regions [8]. In specific nations like Germany, the prevalence among children aged 12 has been documented at rates as high as 28.7% [9]. A contemporary umbrella review corroborated that MIH represents a growing international health challenge for pediatric dentistry, highlighting a critical necessity for standardized, evidence‐based treatment strategies [10]. Furthermore, new clinical data suggests that risk factors such as prenatal complications, early childhood antibiotic use, and systemic illnesses play a major role in the development of MIH [4, 10]. To ensure early identification of the condition, current clinical guidelines stress the importance of meticulously evaluating the morphology of defects and the clarity of enamel opacity margins [11].

Enamel hypomineralization resulting from fluorosis may present in either localized or widespread configurations, frequently appearing as linear, diffuse, or patchy white opacities that lack distinct margins. Furthermore, individuals with amelogenesis imperfecta often exhibit more generalized hypoplastic or hypomineralized defects. Among pediatric patients, the most prevalent hypomineralization disorder is MIH, primarily impacting the first permanent molars and often involving the permanent incisors [12]. Data indicates a global combined prevalence of approximately 14.2% for MIH, which also shows a correlation with hypomineralization observed in second primary molars [9, 13]. The clinical presentation of MIH is diverse, ranging from minor discolored spots to severe posteruptive breakdown (PEB). PEB can progress rapidly, leading to increased sensitivity, patient discomfort, and significant restorative challenges [14]. Enamel loss due to PEB is typically identified by a jagged, irregular transition between affected and healthy enamel, whereas hypoplasia‐related loss usually features smooth, rounded borders. Although the intensity of hypomineralization is generally lower in affected incisors than in molars, the esthetic impact remains a significant clinical concern.

### 3.2. Discoloration

The presence of anterior tooth staining is a major esthetic priority for young patients, significantly impacting their psychological health and overall well‐being [15]. Clinical management of enamel discolorations is complex due to their multifaceted origins, which frequently diminish the esthetic appeal of a patient′s smile. Several mechanisms facilitate the alteration of color within hypomineralized enamel; primarily, the inherent reduction in mineral density increases tissue porosity, heightening the tooth′s vulnerability to staining [16].

• Elevated porosity: Hypomineralized enamel zones exhibit significantly higher porosity compared to healthy enamel. These microscopic voids can capture and retain chromogens from tobacco, beverages, and food, resulting in progressive darkening over time [17].
• Structural surface irregularities: The nonhomogeneous and uneven surface characteristic of hypomineralized enamel provides ideal environments for dental biofilm accumulation. This plaque layer, composed of bacteria and metabolic byproducts, contributes to the development of surface stains [18].
• Permeability to external agents: Because hypomineralized enamel is typically softer and more prone to physical damage, extrinsic staining agents from a patient′s diet can penetrate the enamel structure more easily, leading to deep‐seated discoloration [19].
• Loss of protective barrier: Enamel serves as a natural shield for the underlying dentin; however, when the enamel is mineral‐deficient, it fails to provide sufficient insulation. This allows internal dentin discolorations to become visibly apparent through the translucent enamel surface [20].
• Modified optical characteristics: The altered chemical and structural composition of hypomineralized enamel changes how light interacts with and reflects off the tooth surface. This often produces a dull, lackluster appearance that intensifies the visual perception of the underlying discoloration [21].

Staining in mineral‐deficient enamel can manifest in various clinical forms, such as white, yellow, or brown patches across the teeth [22]. The specific coloration fluctuates depending on the unique properties of the staining agents involved and the extent of the mineral deficiency [21]. A review by Inchingolo et al. emphasized that the severity and specific hue of the resulting discoloration are often correlated with both the protein concentration and the degree of porosity within the hypomineralized enamel [22].

### 3.3. Treatments

Clinically addressing hypomineralization is often complicated by the swift development of dental caries, the difficulty of securing adequate patient cooperation in pediatrics, and the high failure rate of conventional dental restorations [5]. Effectively managing this condition requires a dual focus on both restorative needs and esthetic improvements [10]. In this context, we examine various clinical strategies designed to resolve the esthetic challenges associated with mineral deficiencies. A diverse array of treatment modalities has been developed to improve appearance, including macroabrasion, microabrasion, peroxide‐based bleaching, porcelain veneers, composite laminates, and the etch–bleach–seal protocol.

#### 3.3.1. Comparative Efficacy and Patient Comfort

When compared to conventional conservative interventions, such as peroxide‐based bleaching or microabrasion, the etch–bleach–seal method provides an optimal combination of clinical effectiveness, minimal invasiveness, and high patient comfort. Although microabrasion successfully addresses superficial enamel imperfections, it is frequently inadequate for resolving deeper internal discolorations [23]. Furthermore, while peroxide bleaching is effective, it is often linked to postoperative sensitivity, particularly in pediatric cases [24]. In contrast, the etch–bleach–seal protocol facilitates focused removal of deep stains, preserves maximum tooth structure, and has demonstrated exceptional tolerance and high satisfaction rates among children due to its rapid and noninvasive application [25, 26]. Recent investigations have also examined alternatives like the application of nanohydroxyapatite alongside resin infiltration to strengthen and stabilize enamel affected by MIH, showing encouraging outcomes in both structural and esthetic enhancement [27].

Among the diverse techniques available for enhancing dental esthetics, whitening stands out as a prominent option, primarily employing agents that release peroxide ions [22]. These bleaching materials can be administered through professional in‐office applications or via structured home‐based protocols using lower concentrations of the active agent. Systematic whitening procedures are commonly utilized to achieve a comprehensive esthetic improvement across the anterior dentition [10]. However, despite the clinical efficiency of peroxide‐based compounds, they are frequently associated with the development of dentinal hypersensitivity. As a result, nonperoxide alternatives have been introduced to address enamel discolorations while minimizing these adverse effects [28].

Another frequently suggested approach involves microabrasion, utilized either as a primary treatment or in combination with whitening protocols. Initially developed by Croll, this technique is particularly productive for resolving superficial enamel discolorations or surface‐level defects [29]. It is predominantly indicated for intrinsic staining caused by fluorosis, amelogenesis imperfecta, and enamel hypoplasia [23]. Although this procedure requires the intentional removal of some enamel, it remains significantly more conservative than the extensive surface reduction necessary for porcelain veneers. Additionally, microabrasion and bleaching offer the clinical benefit of being appropriate for use on immature, partially erupted permanent teeth [25].

Definitive restorative solutions like facial veneers are generally deferred until full dental eruption is complete and the gingival margins have reached stability. Consequently, permanent interventions for enamel stains are often delayed until the mid‐to‐late adolescent years. This delay occurs despite the fact that significant concerns regarding dental esthetics frequently emerge much earlier, particularly when discolored teeth are only partially visible during eruption.

Even among very young patients, the psychological impact of anterior tooth discoloration can be quite significant. Consequently, there is a critical need for effective clinical interventions that are appropriate for young permanent teeth, which often feature large pulp chambers, incomplete root development, and partial eruption. To address these specific clinical challenges, the etch–bleach–seal technique has emerged as a highly conservative strategy for managing yellow‐brown intrinsic enamel stains [26].

### 3.4. Rationale for Chosen Intervention

Owing to the young age of the patient and the intrinsic nature of the enamel staining, the etch–bleach–seal method was prioritized over more aggressive restorative interventions to ensure maximum tooth structure preservation. Sodium hypochlorite was specifically chosen as the bleaching agent due to its robust oxidizing capability and its effectiveness in dissolving organic protein matrices within enamel defects.

A small, nonrandomized clinical case series conducted by Prud′homme et al. has previously documented the application of this protocol in pediatric patients with MIH‐related opacities, demonstrating its clinical feasibility and notable esthetic improvements [26]. Nevertheless, because it is a noncomparative case series without a control group and with only three reported MIH cases, this study still represents low‐level evidence, and its outcomes should be interpreted with caution.

### 3.5. Etch–Bleach–Seal Technique

The etch–bleach–seal method, originally introduced by Wright, requires a specific armamentarium including a rubber dam for isolation, a rubber cup, pumice powder, 37% phosphoric acid, 5% sodium hypochlorite, a cotton applicator, and dental sealant [26]. Rubber dam isolation is generally recommended to provide optimal moisture control during adhesive procedures. However, in pediatric clinical settings, alternative isolation methods such as cotton rolls may occasionally be used when patient cooperation is limited, as in the present case. This approach is particularly advantageous for pediatric dentistry due to its focus on maximal tissue preservation. The technique has been specifically recommended for managing yellowish‐to‐brownish enamel opacities [30].

What makes this method distinctive is its targeted ability to neutralize the specific discoloration within the defect. The incorporation of sodium hypochlorite provides significant advantages over peroxide‐based alternatives, particularly for eliminating stains in localized hypomineralized lesions of young permanent teeth. Sodium hypochlorite, the chosen bleaching agent, has an extensive clinical history; it is a staple in endodontic procedures for removing organic debris from root canals and also serves as an effective antimicrobial agent [31]. Contemporary clinical evidence further supports the efficacy of this strategy for anterior teeth, offering a balance between esthetic enhancement and the maintenance of tooth structure [26, 32].

### 3.6. Potential Long‐Term Effects of Sodium Hypochlorite

Sodium hypochlorite is extensively utilized in clinical dentistry as both a disinfecting and bleaching agent and is generally regarded as safe when administered topically in controlled, low concentrations [31]. Within the specific framework of the etch–bleach–seal technique, its short‐term application is intended to target and eliminate organic matter inside hypomineralized enamel without causing substantial damage to the underlying dental architecture [30, 31]. Although the available clinical reports do not suggest adverse effects on the structural integrity or hardness of developing teeth under these conditions, the current evidence is limited and predominantly based on case reports and small clinical series. Consequently, more extensive, longitudinal research is needed to fully clarify the implications of repeated or prolonged exposures.

The potent oxidizing capacity of sodium hypochlorite allows it to fragment and remove smaller degraded molecules, which may contribute to the observed improvements in the appearance of discolored lesions associated with hypomineralization in the existing low‐level clinical literature [30]. A vital secondary phase of this bleaching protocol involves the infiltration of the hypomineralized areas with resin. This step is intended to block future chromogens from entering the porous enamel structure, thereby helping to reduce the risk of subsequent restaining. These procedures, which seek to simultaneously improve optical properties and provide some protection against rediscoloration, offer promising but preliminary avenues for further clinical research in this field [21].

### 3.7. Choice of Acid and Enamel Preparation

The preference for phosphoric acid in this protocol stems from its widespread availability in dental settings and its comparatively milder interaction with enamel when measured against 16% hydrochloric acid [33]. Over several decades, 37% phosphoric acid—a staple agent for resin bonding procedures—has proved highly effective at conditioning enamel crystals and increasing tissue permeability. Consequently, the etch–bleach method relies on standard, readily accessible dental materials that have a long‐standing record of clinical safety and effectiveness [34].

Managing enamel with elevated organic concentrations through traditional bonding protocols is often difficult, as the organic matrix acts as a physical obstruction to effective etching [30]. Research has indicated that the application of sodium hypochlorite can efficiently strip these proteins from the surface of enamel crystallites. Moreover, utilizing sodium hypochlorite as a pretreatment has been shown to amplify the acid′s ability to condition the surface, thereby optimizing the environment for successful resin adhesion [35]. A study conducted in 2023 further confirmed that the synergistic use of sodium hypochlorite and phosphoric acid etching significantly upgrades bond strength while maintaining enamel roughness within clinically acceptable limits [36].

The etch–bleach–seal technique utilizes readily accessible materials with a high safety profile and can be effectively administered to young permanent teeth. This clinical strategy remains highly relevant even for permanent incisors that have only partially erupted, providing significant advantages for both older children and early adolescents [26, 30]. The etch–bleach–seal method represents a conservative treatment alternative for yellowish‐to‐brownish hypomineralized enamel, with case reports and small clinical series suggesting favorable short‐term esthetic outcomes. However, the supporting evidence remains limited in scope and largely observational, and robust data on long‐term esthetic stability are still lacking. When managing enamel discolorations, it is generally advisable to consider conservative treatment modalities before resorting to interventions that require substantial removal of the enamel surface.

The clinical variation of the etch–bleach–seal technique described by Dr. Jeanette MacLean is based on a non–peer‐reviewed educational video resource [33], which we adopted for its practical guidance in day‐to‐day clinical application. This protocol is conceptually consistent with the original etch–bleach–seal technique reported in the peer‐reviewed literature [34] but should be interpreted in the context of its non–peer‐reviewed origin.

### 3.8. Clinical Implications

The application of the etch–bleach–seal technique is highly feasible for young permanent teeth, utilizing standard materials that maintain an excellent safety profile [26]. This clinical approach remains particularly relevant for treating partially erupted permanent incisors, offering notable therapeutic benefits for both school‐aged children and young adolescents [30].

As established in earlier dental literature, Wright was the first to provide a comprehensive description of the etch–bleach–seal protocol [26]. Further validation of this method was provided by Namdev et al., who investigated its efficacy in three patients presenting with mild to moderate dental fluorosis. Their research team successfully employed a combination of microabrasion and the etch–bleach–seal procedure to target and eradicate fluorotic stains. The results of their study were highly favorable, further establishing the clinical effectiveness of this integrated approach [36].

In a separate in vitro randomized controlled trial, Gandhi et al. evaluated how deproteinization pretreatment influences the penetration of resin sealants into enamel affected by MIH [25]. The study utilized 31 extracted MIH‐affected teeth, which were categorized into three distinct experimental groups: a control group (standard etching followed by fissure sealing), Treatment Group 1 (5% sodium hypochlorite application, followed by etching and fissure sealing), and Treatment Group 2 (application of 5% NaOCl and fissure sealing, excluding the etching step). The findings revealed no significant difference in the quality of resin tags between the conventional sealing method (control) and the bleach–etch–seal protocol (Treatment 1). Furthermore, applying NaOCl alone without the etching phase prior to sealing did not provide any supplementary benefits. The authors highlighted a substantial probability of encountering substandard sealant tags in MIH‐affected enamel, regardless of the specific intervention applied [25].

A small, noncomparative clinical case series in MIH patients (three cases), representing low‐level evidence by Prud′homme et al., demonstrated varied therapeutic results and illustrated the potential and limitations of the etch–bleach–seal approach [26]. In the first case, an 11‐year‐old male presenting with yellow‐brown lesions on his maxillary central incisors underwent treatment using exclusively the etch–bleach–seal protocol. This intervention successfully transitioned the discolorations into white defects, significantly enhancing the patient′s esthetic appearance and satisfaction. In the second case, a 12‐year‐old female with yellow‐brown imperfections on three mandibular incisors received a dual‐stage strategy—combining microabrasion with the etch–bleach–seal method—which resulted in moderate clinical improvement. Finally, a 7‐year‐old female with a deep yellow‐brown lesion required a more intensive integrated protocol involving microabrasion, etch–bleach, and resin infiltration. While this combined approach reduced the color contrast, it did not result in complete resolution of the defect. Taken together, this case series provides useful clinical insight, but, due to its noncomparative design and small sample size, it still represents a low level of evidence.

Further low‐level clinical evidence was provided by Asthana et al., who successfully utilized the etch–bleach–seal technique to manage two cases of mild dental fluorosis [37]. In addition, Singhal et al. reported a randomized clinical trial comparing different bleaching and microabrasion protocols in children with nonpitted fluorotic stains, including the etch–bleach–seal method [34]. Although this study offers valuable preliminary comparative data, it does not specifically address MIH‐related hypomineralization, and the authors themselves concluded that larger randomized controlled clinical trials are required to determine the most effective treatment modality and to strengthen the overall level of evidence.

### 3.9. Long‐Term Effects and Considerations

Although the immediate clinical results of the etch–bleach–seal procedure in this case were highly positive, the prospective long‐term impact on the enamel′s structural composition remains a subject of ongoing clinical debate. The repetitive use of sodium hypochlorite and phosphoric acid, while highly efficient for deproteinization and whitening, could potentially modify surface hardness or heighten vulnerability to wear if the sealing process is not meticulously performed. Existing dental literature indicates that these risks are substantially mitigated when followed by thorough resin infiltration; however, there is still a notable lack of longitudinal clinical research to definitively assess factors such as color stability, enamel integrity, and long‐term resistance to subsequent staining [34, 36, 38]. Until more comprehensive data become available, it is advised that clinicians perform regular follow‐up examinations on treated teeth and exercise professional caution when applying this protocol, particularly in very young pediatric patients.

### 3.10. Scientific Rationale

The clinical success observed in this case is fundamentally rooted in the biochemical interaction between sodium hypochlorite and the hypomineralized enamel matrix. By effectively dissolving the trapped organic proteins that characterize MIH‐affected enamel, the protocol transforms the porous, discolored zone into a receptive substrate for resin infiltration. This dual action—chemical oxidation followed by structural sealing—provides a robust scientific rationale for the immediate esthetic improvement and the prevention of future chromogenic ingress. Consequently, the etch–bleach–seal technique serves as a biologically sound and minimally invasive alternative for pediatric patients.

### 3.11. Patient Perspective

The patient and her parents reported a very positive experience. They were particularly pleased that the procedure was noninvasive and did not require “drilling” or local anesthesia, yet provided a significant esthetic improvement that boosted the child′s confidence.

### 3.12. Primary Takeaway Lesson

The etch–bleach–seal protocol may represent a promising, minimally invasive therapeutic option for addressing yellow‐brown hypomineralized enamel defects in selected pediatric patients. This method has the potential to combine meaningful esthetic improvements with preservation of the patient′s natural tooth structure. In light of the currently available low‐level evidence (mainly case reports and small clinical series), this approach can be considered one conservative option within a broader spectrum of management strategies for developmental enamel abnormalities in young patients, rather than as a routinely prioritized standard of care.

From an evidence‐based standpoint, the current literature on the etch–bleach–seal technique encompasses a spectrum of study designs with predominantly low methodological strength. The majority of reports consist of single case reports and a small clinical case series documenting esthetic improvement in individual patients with MIH or fluorosis [26, 38]. In addition, one in vitro randomized controlled trial has evaluated the influence of deproteinization pretreatment on resin sealant penetration in extracted MIH‐affected teeth [25]. To date, only one randomized clinical trial has compared different minimally invasive bleaching and microabrasion protocols, including the etch–bleach–seal method, in children with nonpitted fluorotic stains rather than MIH [34]. Collectively, these heterogeneous and mostly low‐level studies provide useful preliminary insights but are insufficient to establish robust, high‐level clinical recommendations for the use of the etch–bleach–seal technique in MIH.

## 4. Conclusion

In conclusion, the etch–bleach–seal method is a simple, conservative, and cost‐effective approach for managing enamel hypomineralization, and it can yield favorable short‐term clinical and esthetic results in pediatric patients. This single case report, combined with a narrative review of 35 articles, suggests that the etch–bleach–seal technique may represent a valuable minimally invasive option in selected MIH cases. However, the current evidence base is predominantly derived from single case reports, a small clinical case series, a few narrative reviews, and one preliminary randomized trial, all of which correspond to a relatively low level of evidence. While the immediate outcomes are encouraging, the lack of robust comparative studies, objective outcome measures, and long‐term data necessitates caution in interpreting these findings. Therefore, future research should include larger, well‐designed randomized controlled clinical trials, broader case series with standardized outcome criteria, and complementary in vitro studies to validate the long‐term stability of this method and its impact on enamel integrity over time.

### 4.1. Strengths and Limitations

A major strength of this case lies in its real‐world application of the etch–bleach–seal technique, supported by literature and adapted to a pediatric patient. However, a limitation is the absence of long‐term follow‐up beyond 3 months.

## Author Contributions

**Matine Gharavi:** conceptualization, investigation (case management), data curation, writing – original draft preparation. **Sara Tavassoli-Hojjati:** methodology, supervision, validation, writing – review and editing. **Zahra Abdolahpour:** literature review/resources, visualization (figure preparation), writing – review and editing.

## Funding

No funding was received for this manuscript.

## Consent

Written informed consent was obtained from the patient′s legal guardians for publication of this case report and accompanying clinical images.

## Conflicts of Interest

The authors declare no conflicts of interest.

## Data Availability

The data that support the findings of this study are available on request from the corresponding author. The data are not publicly available due to privacy or ethical restrictions.
